# Observations From a Mouse Model of Forebrain Voa1 Knockout: Focus on Hippocampal Structure and Function

**DOI:** 10.3389/fncel.2019.00484

**Published:** 2019-11-21

**Authors:** Ke Ma, Na-Ryum Bin, Shan Shi, Hidekiyo Harada, Yoh Wada, Ge-Hong-Sun Wada, Philippe P. Monnier, Shuzo Sugita, Liang Zhang

**Affiliations:** ^1^Department of Pediatric Outpatient, The First Hospital of Jilin University, Jilin, China; ^2^Krembil Research Institute, University Health Network, Toronto, ON, Canada; ^3^Division of Biological Science, Institute of Scientific and Industrial Research, Osaka University, Osaka, Japan; ^4^Department of Biochemistry, Faculty of Pharmaceutical Sciences, Doshisha Women’s College, Kyoto, Japan; ^5^Department of Ophthalmology, University of Toronto, Toronto, ON, Canada; ^6^Department of Physiology, University of Toronto, Toronto, ON, Canada; ^7^Department of Medicine, University of Toronto, Toronto, ON, Canada

**Keywords:** V-ATPase, spatial memory, glutamate transamination, neuronal degeneration, synapse, brain slice, EEG, conditional knock out mice

## Abstract

Voa protein is a subunit of V-ATPase proton pump which is essential to acidify intracellular organelles including synaptic vesicles. Voa1 is one of the four isoforms of Voa family with strong expression in neurons. Our present study was aimed to examine the role of Voa1 protein in mammalian brain neurons. To circumvent embryonic lethality, we generated conditional Voa1 knockout mice in which Voa1 was selectively deleted from forebrain pyramidal neurons. We performed experiments in the Voa1 knockout mice of ages 5–6 months to assess the persistent effects of Voa1 deletion. We found that the Voa1 knockout mice exhibited poor performance in the Morris water maze test compared to control mice. In addition, synaptic field potentials of the hippocampal CA1 region were greatly diminished in the Voa1 knockout mice when examined in brain slices *in vitro*. Furthermore, brain histological experiments showed severe degeneration of dorsal hippocampal CA1 neurons while CA3 neurons were largely preserved. The CA1 neurodegeneration was associated with general brain atrophy as overall hemispheric areas were reduced in the Voa1 cKO mice. Despite the CA1 degeneration and dysfunction, electroencephalographic recordings from the hippocampal CA3 area revealed aberrant spikes and non-convulsive discharges in the Voa1 knockout mice but not in control mice. These hippocampal spikes were suppressed by single intra-peritoneal injection of diazepam which is a benzodiazepine GABA_A_ receptor enhancer. Together these results suggest that Voa1 related activities are essential for the survival of the targeted neurons in the dorsal hippocampal CA1 as well as other forebrain areas. We postulate that the Voa1 knockout mice may serve as a valuable model for further investigation of V-ATPase dysfunction related neuronal degeneration and functional abnormalities in forebrain areas particularly the hippocampus.

## Introduction

Vacuolar H^+^-ATPases or V-ATPases are multi-subunit proton pumps that acidify a wide array of intracellular organelles including synaptic vesicles (Moriyama and Futai, 1990) and are crucial for many fundamental processes including endocytosis, signaling molecule processing, protein sorting, trafficking, lysosomal as well as autophagosomal enzyme activation (Forgac, 2007; Cotter et al., 2015). In general, V-ATPases consist of a Vo proton-translocation domain and a V1 pump domain and two associated proteins Ac45 and ATP6AP2. The largest (~110 kDa) subunit of the Vo domain, Voa, is an integral membrane protein with four isoform genes (a1–a4) identified in human, mouse and *Drosophila*. While Voa1, Voa2, and Voa3 are widely expressed, Voa1 is more strongly expressed in the brain, whereas Voa3 is primarily expressed in osteoclasts (Perin et al., 1991; Nishi and Forgac, 2000; Toyomura et al., 2000).

The proteolipid pore-forming Vo domain of V-ATPases is thought to be required for membrane fusion downstream of Soluble N-ethylmaleimide-sensitive factor attachment protein receptor (SNARE) complex (Peters et al., 2001; Hiesinger et al., 2005; Peri and Nüsslein-Volhard, 2008; Saw et al., 2011; Wang et al., 2014). For example, loss-of-function mutations in a neuron-specific Voa subunit (Vha100–1) exhibit defects in a late stage of synaptic exocytosis, which suggests that Vha100–1 is a regulator of synaptic vesicle fusion efficiency downstream of SNARE-dependent vesicle priming in *Drosophila* (Hiesinger et al., 2005). In mouse hippocampal neurons, Voa1-containing V-ATPase does not play a direct role in synaptic vesicles fusion but modulates neurotransmitter release upstream of docking by facilitating fusion of fully acidified and neurotransmitter-loaded synaptic vesicles (Bodzeta et al., 2017). While Voa1 over-expression does not produce significant alterations in glutamatergic synaptic transmission in rat hippocampal neurons (Chanaday and Kavalali, 2018), Voa1 dysfunction is implicated in neurological diseases and relevant models (Colacurcio and Nixon, 2016). In particular, impaired glycosylation and instability of Voa1 leading to deficiencies in lysosomal V-ATPase assembly are recognized in a mouse model of Alzheimer Disease (Lee et al., 2015). Misrouted V-ATPase Voa1 is a major cause of dysregulated lysosomal acidification in neurons of a mouse model of severe infantile neuronal ceroid lipofuscinoses (Bagh et al., 2017). Together these findings call further investigation into the role of Voa1 protein in mammalian brain neurons. Our present study was aimed to provide more information in this area with a particular focus on the hippocampus.

Global knockout of this gene was known to be embryonic lethal (Dickinson et al., 2016). Therefore, we generated a conditional Voa1 knockout (Voa1 cKO) model using CaMK2-Cre mice (Tsien et al., 1996) to selectively delete Voa1 in pyramidal neurons of forebrain areas including the hippocampus. By examining Voa1 cKO mice of ages 5–6 months, we tested the hypothesis that chronic Voa1 deletion may cause pronounced structural and functional abnormalities in targeted hippocampal neurons.

## Materials and Methods

### Animals

All mice were maintained in a vivarium on a 12-h light on/off cycle and at a temperature between 22 and 23°C. Mice were housed in standard cages with food and water *ad libitum*. All experiments performed were reviewed and approved by the animal care committee of the University Health Network in accordance with the Canadian Guidelines for Animal Care.

Voa1 flox mice (Atp6v0a1_tm1c_H11) were obtained from the Center for Phenogenomics in Toronto. Diagram of the floxed allele in comparison with WT allele is shown in Supplementary Figure S1A. The genetic background of ES cells to generate the Voa1 flox mice is C57BL/6N. We obtained C57BL/6 mice with CaMKIIalpha-Cre (Tsien et al., 1996) from the Jackson Laboratory (#005359; B6.Cg-Tg(CaMKIIalpha-cre)T29-1Stl/J) to generate conditional knockout mice. Genotyping of each animal was performed using PCR by extracting genomic DNA from the tail. Specifically, mouse tails were collected and genomic DNA was obtained by an alkaline lysing method where the samples were lysed with 50 mM NaOH at 95°C for 2 h. Then 0.5 M Tris-HCl (pH 8.0) was added to neutralize pH. The supernatant was directly used in PCR for genotyping. To detect the difference between wild type vs. floxed allele, PCR was performed using a forward primer 5′-ACCTGGTGTATTCCATTCACTC-3′ and a reverse primer 5′-TTCTTCTGCCCCAGGATGATG-3′, which are located on the upstream and downstream of 5′ loxP site of exon 3 (see the diagram in Supplementary Figure S1A). For Cre recombinase PCR, a forward primer of 5′-GAACCTGATGGACATGTTCAGG-3′ and a reverse primer of 5′-AGTGCGTTCGAACGCTAGAGCCTGT-3′ were used. For all experiments, Voa1 flox mice without CaMKIIalpha-Cre were used as controls.

### Voa1 Knockout in Cultured Hippocampal Neurons

Hippocampi were dissected out from postnatal day 0–1 Voa1 floxed mice of both sexes. Neurons were prepared and cultured as described previously (Kavalali et al., 1999) with minor modifications. Briefly, neurons were dissociated by treatment with trypsin (10 mg/ml) and DNase (0.25 mg/ml) for 10 min at 37°C, triturated with a 200 μl pipette tip and then plated onto 12 mm coverslips coated with Matrigel (BD Biosciences) and poly-L-ornithine. A plating density of two hippocampi per six coverslips was used. Culture media consisted of minimal essential media, 5 mg/ml glucose, 0.1 mg/ml transferrin, 0.5 mM GlutaMAX, 5% iron supplemented calf serum (Hyclone), 2% B-27 supplement, 1 μM cytosine arabinoside, 50 units/ml of penicillin, and 50 μg/ml of streptomycin. Cultures were maintained at 37°C in a humidified incubator gassed with 95% air and 5% CO_2_. Cells were infected with lentiviral particles that co-expressed GFP and blasticidin S resistance gene (bsr, control) or GFP and Cre recombinase gene (KO) on day *in vitro* 1 (DIV 1). Whereas control neurons easily survived more than DIV 21, KO neurons started showing signs of deterioration on DIV 13 and completely degenerated on DIV 15. Therefore, both control and KO neurons were harvested on DIV 13 with a cell scraper and subjected to Western blot analysis in which each lane of SDS-PAGE was loaded with 5 μg homogenates.

### Voa1 Expression in Wild Type Mice

Immunofluorescence microscopy in brain sections was used to examine Voa1 expression. The mouse was anesthetized by sodium pentobarbital (70 mg/Kg, intra-peritoneal injection) and transcardially infused with PBS and then with 4%PFA. The brain was removed and further fixed in 4%PFA for >24 h and then with a hypertonic (with 30% sucrose) formalin solution for >24 h. Coronal sections of 30 μm thickness were obtained and then incubated with blocking buffer (PBS containing 0.1% Triton X-100 and 2% BSA) for 45 min. Voa1 antibody (IgY, chicken monoclonal antibody generated from Dr. Yoh Wada lab of Osaka University, Japan; see also Sun-Wada et al., 2011) was added to the blocking buffer at 1:100 dilution. The sections were incubated with the antibody at 4°C overnight. Then, they were washed with PBST (PBS containing 0.1% of Tween 20) three times and incubated with Alexa Fluor goat anti-chick antibody (1:1,000) in dark for 1 h. Brain sections similarly stained by the goat anti-chick antibody alone (without the chicken monoclonal Voa1 antibody) were used to control non-specific signals. For subsequent DAPI staining, the samples were washed with PBS three times before and after 5 min-incubation with 0.1 mg/ml DAPI (product# ab228549; abcam.com). The stained samples were mounted on the glass slide with fluoromount G (Southern Biotech, Birmingham, AL, USA) and covered by a cover slip. Images were obtained using a fluorescent microscope (Olympus BX61) and Olympus DP70 digital camera.

### Morris Water Maze Test

We used a maze protocol with 9-day trials and two-probe tests (Bin et al., 2018). Mice underwent visible platform trials for 3 days and then hidden platform trials for 6 days (90 s per trial and four trials per day). The inter-trial intervals were roughly 10–15 min. For visible platform trials, if the mice were unable to find the platform within 90 s, they were guided to the platform by the experimenter’s hand. For hidden platform trials, the procedure was identical to visible platform trials with the exception that the platform was submerged underwater at a depth of 1.5 cm and the platform location was changed. The distances and latencies individual mice needed to find the platform during visible and hidden platform trials were measured. For the probe tests, the times the mice spent in searching the pool quadrant where the platform was previously located were measured. We used Micro-Manager 2.0 (Open Source Microscopy Software) for recording and ImageJ (National Institute of Health, Bethesda, MD, USA) software for analysis in the Morris water maze test.

### Electrophysiological Recordings in Brain Slices

Brain slice preparation and *in vitro* recordings were done as previously described (Bin et al., 2018; Song et al., 2018a). Briefly, the mouse was anesthetized by sodium pentobarbital (70 mg/Kg, intra-peritoneal injection) and transcardially infused with cold, dissection-only artificial cerebrospinal fluid (ACSF) before brain dissection. Brain slices of 0.4 mm thickness were obtained using a vibratome (Leica VT1200) in ice-cold dissection-only ACSF. Slices encompassing dorsal hippocampal areas (roughly corresponding to Bregma −1.46 to −2.18 mm) were obtained *via* coronal sections. Slices encompassing ventral hippocampal areas were obtained *via* horizontal sections from the basal brain as the ventral hippocampus can be obtained at the transverse plane in these slices. The first three horizontal slices with clearly recognizable ventral hippocampal areas were collected for recordings. After vibratome section, slices were maintained in oxygenated (5%CO_2_–95%O_2_) standard ACSF at room temperature (21–22°C) for 1–6 h before recordings. The dissection-only ACSF contained (in mM): sucrose 300, KCl 3.5, CaCl_2_ 0.5, MgCl_2_ 6, HEPES 5 and glucose 20 (pH adjusted to 7.4). The standard ACSF contained (mM): NaCl 125, KCl 3.5, NaH_2_PO_4_ 1.25, CaCl_2_ 2, MgSO_4_ 1.3, NaHCO_3_ 25 and glucose 10 (pH 7.35–7.4 when aerated with 5%CO_2_–95%O_2_).

All recordings were done in a submerged chamber where each slice was perfused with oxygenated (5%CO_2_–95%O_2_) standard ACSF at room temperature. Extracellular recording electrodes were made of thin-wall glass tubes (World Precision Instruments, Sarasota, FL, USA) and filled with a solution with 150 mM NaCl and 2 mM HEPES (pH 7.4; resistance of 1–2 MΩ). Local field potentials were recorded using an Axoclamp 2B amplifier with input frequencies set between 0–1,000 Hz. Signals were amplified by 1,000 times and then digitized at 10,000 Hz (Digidata 1440, Molecular Devices/Axon Instruments, Sunnyvale, CA, USA). A bipolar electrode, made of polyimide-insulated stainless steel wires (outer diameter 0.1 mm; Plastics One, Ranoake, VA, USA), was placed in the CA2 striatum radium area to stimulate the Schaffer collateral-CA1 pathway. Constant current pulses (0.1 ms duration, intensities of 10–150 μA) were generated by a Grass stimulator and delivered through a photoelectric isolation unit every 20 s (S88, Natus Neurology Incorporated—Grass Products, Warwick, RI, USA). Data acquisition, storage and analysis were completed using PClamp software (version 10, Molecular Devices).

Synaptic field potentials were evoked by paired stimuli (interval 25 ms) with incrementing intensities of 10–150 μA (10 μA per step) and recorded from the CA1 striatum radium area. Four consecutive responses evoked by a given intensity were averaged for measurements of presynaptic volley and synaptic potential amplitudes.

### Brain Histology

Brain histological assessments were performed as previously described (Bin et al., 2018; Song et al., 2018a). Briefly, the mouse was anesthetized by sodium pentobarbital and transcardially infused with the dissection-only ACSF and then with 10% neutral buffered formalin solution. The brain was removed and further fixed in a hypertonic (with 20% sucrose) formalin solution for ≥24 h. Cryostat coronal sections of 25 μm thickness were obtained and stained. Images were obtained using a slide scanner (Aperio digital pathology slide scanner AT2, Leica) at 20× magnification and analyzed using ImageScope (Leica) and ImageJ software. Hemispheric area, hippocampal area including CA1 and CA3 cell body layers were measured from sections corresponding Bregma −1.7 mm to −1.94 mm and Bregma −3.16 mm to −3.4 mm. The densities of CA1 and CA3 cell body layers were measured from middle CA1 and CA3 regions (200 μm in length). Brightness and contrast of grayscale images were adjusted to distinguish cell body layers from adjacent dendritic layers. The areas (pixel numbers) of cell body layers were measured from adjacent three sections for each mouse.

### Electroencephalographic (EEG) Recordings

Electrode implantation and electroencephalographic (EEG) recordings were performed as previously described (Bin et al., 2017; Song et al., 2018b). All electrodes were made with polyimide-insulated stainless-steel wires (outside diameter 0.1 mm, Plastics One). Two pairs of bipolar electrodes were implanted bilaterally in middle-ventral hippocampal CA3 areas (Bregma −2.9 mm, lateral 2.5 mM and depth 3.0 mm: Franklin and Paxinos, 1997). A reference electrode was positioned into a frontal area (Bregma +2.0 mm, lateral 1.0 mm and depth 0.5 mm). We implanted electrodes in the middle-ventral hippocampal CA3 area because dorsal hippocampal areas were decreased in the Voa1 cKO mice and it was difficult to target the dorsal hippocampal CA3 in these mice. Implanted mice were allowed to recover for ≥1 week prior to baseline monitoring. The locations of implanted electrodes were verified by later histological assessments if suitable.

EEG recordings were performed in free-moving mice. EEG signals were collected using a dual-channel AC microelectrode amplifier (model 1800, AM Systems, Sequim, WA, USA) with input frequencies set between 0.1 and 1,000 Hz and amplification by 1,000 times. Data digitization (at 5 KHz), acquisition, storage and analysis were done using the Molecular Devices system as described above. EEG spikes were recognized by intermittent events with large peak amplitudes (≥6 times of standard deviation of background signals), simple or complex spike waveforms, and durations of 30–250 ms (El-Hayek et al., 2013). Spike incidences were measured from 50 to 70 min data segments collected during stable immobility or sleep, as hippocampal spikes manifested in these “inactive” behavioral states and EEG signals were minimally interfered by movement-related artifacts (El-Hayek et al., 2013). An event detection function (threshold search method) of PClamp software was used to detect spikes automatically, and detected events were then visually inspected and false events were rejected.

Diazepam was obtained in a clinically injectable form (Sandoz Canada Inc., Boucherville, QC, Canada) and diluted with saline for intraperitoneal injection (1.5 mg/kg/injection, El-Hayek et al., 2013). Saline injections were used as controls. Data collected within 8 h post diazepam or saline injections were compared in individual mice.

### Statistical Analysis

All statistics were performed using Origin 9 (OriginLab, Northampton, MA, USA) or SPSS (IBM Corporation, Armonk, NY, USA). Data were presented as mean and standard error of mean (SEM) throughout the text and figures. Statistical significance was set at *p* < 0.05. A student’s *t*-test (independent or paired) was used for two group comparisons. A mixed repeated ANOVA was used for comparison between groups. A one-way repeated ANOVA was used for comparison within group.

## Results

Voa1 is widely expressed in the brain of wild type mice including the hippocampus as revealed by immunofluorescent imaging with Voa1 antibody (Supplementary Figure S2). The Voa1 cKO model we generated using CaMKIIα-Cre mice (Tsien et al., 1996) targets selectively forebrain pyramidal neurons particularly those in the dorsal hippocampal CA1. We, therefore, focused on hippocampal structures and functions in the present experiment. Specifically, we conducted experiments in the Voa1 cKO and control mice of ages 5–6 months to determine the persistent effects of Voa1 deletion.

### Impaired Spatial Memory in Voa1 cKO Mice

**Figure 1:**
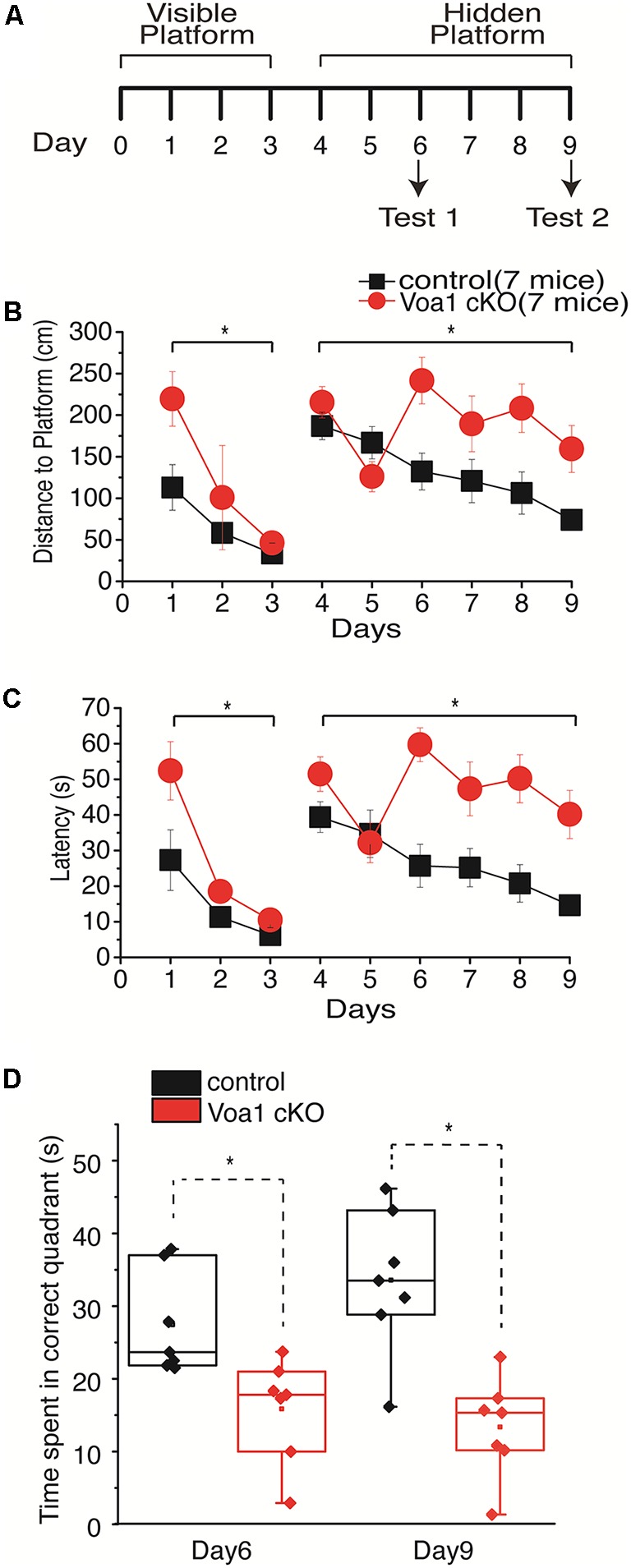
Spatial memory impaired in the Voa1 cKO mice. **(A)** A maze protocol with 3 days of visible platform trials, 6 days of hidden platform trials and two probe tests was used. Swimming distance and time taken to reach the platform during the visible and hidden platform trials were presented in **(B,C)**. For the probe tests, the times spent in the correct quadrant where the platform was previously located were measured and presented in **(D)**. There were significant differences between the Voa1 cKO mice and control mice in the distance and time measures during the visible and hidden platform trials (Mixed repeated ANOVA; see “Results” section) as well as in probe tests (*t*-test). Here and afterwards, group data were presented as means and standard errors of means. Statistical significances (*p* ≤ 0.05) between the Voa1 cKO mice and control mice were denoted by *. Non-significant difference denoted by n.s. Data points in box plots represented the maximal, minimal, 25% and 75% measures of individual groups, and horizontal lines and squares in boxes represented median and mean values. Data and responses collected from the Voa1 cKO or control mice were marked by red or black colors.

We first compared the performances of the Voa1 cKO and wild type control mice in the Morris water maze task. We used a 9-day protocol consisted of 3 days of visible platform trials, 6 days of hidden platform trials and two-probe tests (Bin et al., 2018; Figure 1A). Data were collected from seven Voa1 cKO mice and seven control mice (three female and four male mice in each group).

During the visible platform trials, both the Voa1 cKO and control mice improved significantly in performance over the 3-day trials, as there were day/trial-dependent reductions in swimming distance (*F*_(2,12)_ = 5.048, *p* = 0.026 and *F*_(2,12)_ = 4.813, *p* = 0.029) and time (*F*_(2,12)_ = 20.899, *p* < 0.001 and *F*_(2,12)_ = 4.460, *p* = 0.036) in finding the platform (Figures 1B,C). Significant differences between the Voa1 cKO and control mice were observed with respect to the distance (*F*_(1,12)_ = 6.558, *p* = 0.025) and time (*F*_(1,12)_ = 7.028, *p* = 0.021) traveled to reach the platform (Figures 1B,C).

During the 6-day hidden platform trials, the control mice were improved in both distance (*F*_(5,30)_ = 5.314, *p* = 0.001) and time (*F*_(5,30)_ = 4.468, *p* = 0.004) needed to reach the platform; while the Voa1 cKO mice showed significant improvement in swimming distance (*F*_(5,30)_ = 2.842, *p* = 0.032) but not in time measures (*F*_(5,30)_ = 2.535, *p* = 0.050). There were significant differences between the two groups: the Voa1 cKO mice swam significantly longer distances (*F*_(1,12)_ = 9.464, *p* = 0.01) and spent more time (*F*_(1,12)_ = 21.261, *p* = 0.001) than the control mice to reach the platform (Figures 1B,C).

During the probe test 1 and 2, the Voa1 cKO mice spent significantly less time in the correct quadrant than the controls (*p* = 0.01 and *p* = 0.001; Figure 1D). Together, these observations suggest impaired spatial learning and memory in the Voa1 cKO mice.

### CA1 Synaptic Field Potentials Were Diminished in Voa1 cKO Mice

**Figure 2:**
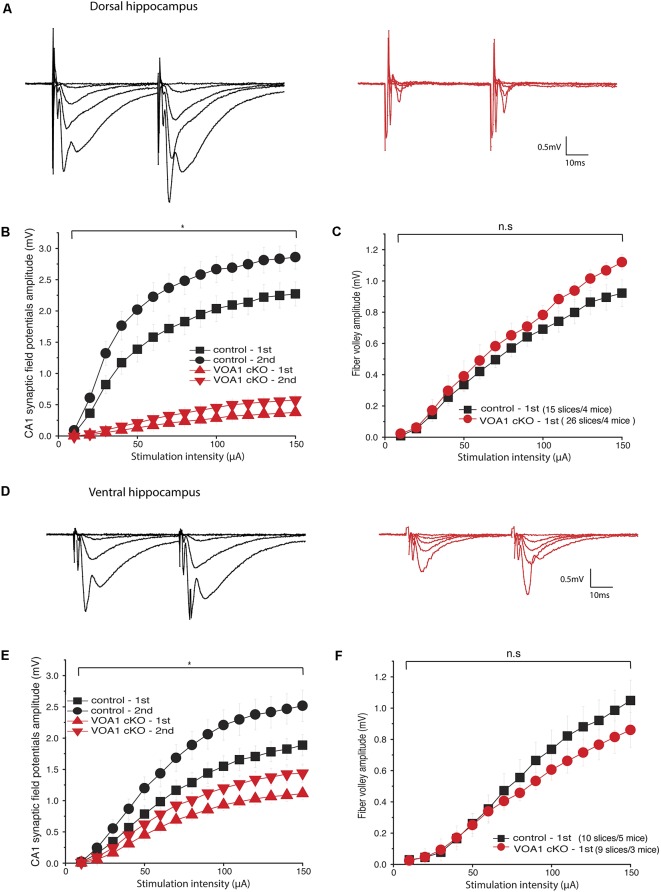
CA1 synaptic field potentials diminished in the Voa1 cKO mice. **(A,D)** Representative traces were collected from the Voa1 cKO mice and the control mice. Dorsal **(A)** and ventral **(D)** CA1 synaptic field potentials were evoked by paired stimulations of the Shaffer collateral pathway at intensities of 10, 20, 30, 50 or 150 μA, respectively. **(B,E)** The amplitudes of 1st and 2nd CA1 field potentials were plotted vs. stimulation intensities. **(C,F)** The amplitudes of presynaptic volleys were plotted vs. stimulation intensities. The synaptic field potentials **(B,E)**, but not the presynaptic volleys **(C,F)**, were significantly different between the Voa1 cKO mice and control mice (Mixed repeated ANOVA; see details in “Results” section).

We next examined hippocampal synaptic activities in the Voa1 cKO mice and focused on the well-characterized Shaffer collateral-CA1 pathway. Extracellular recordings in brain slices were used to assess changes in transmission at a local circuitry level (15–26 slices from four Voa1 cKO or control mice). Synaptic field potentials were recorded from the CA1 striatum radium which is the apical dendritic layer of the CA1 pyramidal neurons. Paired stimulations (25 ms interval) of different intensities were used to examine the overall activity and short-term plasticity of the Shaffer collateral-CA1 pathway. Monitored from dorsal hippocampal areas, CA1 synaptic field potentials of the Voa1 cKO mice were either unrecognizable or seemingly small amplitudes. The overall amplitudes, evoked by the first and second stimulations were greatly diminished as compared to the controls (*F*_(1,39)_ = 104.553, *p* < 0.001 and *F*_(1.39)_ = 142.727, *p* < 0.001; Figures 2A,B).

Despite the great group differences in synaptic field potentials, the amplitudes of presynaptic volleys, which present the overall activity of activated presynaptic fibers of the Schaffer collateral-CA1 pathway, were not significantly different between the Voa1 cKO and control mice (*F*_(1,39)_ = 1.226, *p* = 0.275; Figure 2C). These observations suggest that postsynaptic dysfunction may largely account for the diminished CA1 synaptic potentials in the Voa1 cKO mice, which is consistent with the histological assessment described below.

Monitored from ventral hippocampal areas, CA1 field potentials evoked by the first and second stimulations were also decreased in the Voa1 cKO mice relative to the controls (*F*_(1,17)_ = 10.485, *p* = 0.005 and *F*_(1,17)_ = 13.811, *p* = 0.002); whereas the amplitudes of presynaptic volleys were largely similar between the Voa1 cKO and controls mice (*F*_(1,17)_ = 0.672, *p* = 0.424; Figures 2D–F). Overall, the diminished CA1 field potentials in the Voa1 cKO mice were more pronounced in the dorsal than in ventral hippocampal areas examined (Figures 2B,E). As glutamatergic activities are the predominant component of the CA1 synaptic field potentials, these observations suggest regional alterations of hippocampal CA1 glutamate synapses in the Voa1 cKO mice. The decreased ventral CA1 field potentials might be largely due to alterations in synaptic transmission as Voa1-containing V-ATPase has been suggested to modulate neurotransmitter (glutamate) release in rat hippocampal neurons (Bodzeta et al., 2017).

### Hippocampal CA1 Neurodegeneration in Voa1 cKO Mice

**Figure 3:**
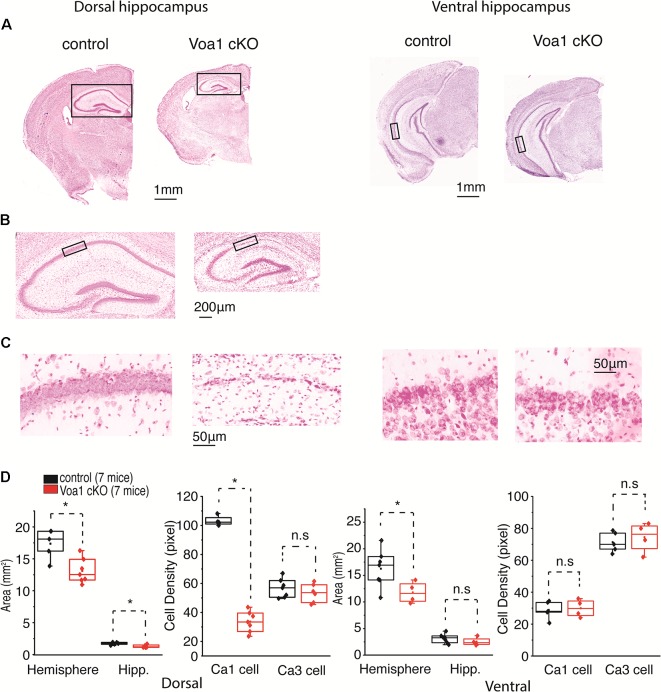
Dorsal hippocampal CA1 neurons degenerated in the Voa1 cKO mice. **(A**–**C)** Images were collected from two control mice and two Voa1 cKO mice. Representative coronal sections encompassing dorsal or ventral hippocampal areas were stained with cresyl violet. Squared hippocampal areas in **(A)** left panels were expanded in **(B)**. Squared CA1 sectors in **(B)** or in **(A)** right panel were enlarged in **(C)** left or right panel. **(D)** Left and **(D)** right, hemispheric and hippocampal areas and densities of CA1 and CA3 cell body layers in coronal sections encompassing dorsal and ventral hippocampal areas respectively. Cell densities measures were made from adjacent three sections for each mouse. Data collected from seven Voa1 cKO mice and seven control mice. Note that hemispheric and dorsal hippocampal areas were decreased in the Voa1 cKO mice relative to the controls (independent *t*-test). Note also that the density of CA1 cell body layer was greatly decreased in the dorsal hippocampus of the Voa1 cKO mice.

We also performed general histological experiments to see whether the above functional assessments are associated with evident neuronal degeneration in the Voa1 cKO mice. Coronal sections (25 μm) were stained with cresyl violet for general morphological assessments. In sections encompassing dorsal hippocampal areas (Bregma −1.7 to −1.94 mm), brain hemispheric and hippocampal areas were decreased in the Voa1 cKO mice (*n* = 7) relative to the control mice (*n* = 7; *p* = 0.006 or 0.008; Figures 3A,D). In addition, degenerated hippocampal CA1 neurons were evident in all Voa1 cKO mice examined and featured with vanished or diminished CA1 cell body layer (Figures 3B,C). The overall CA1 cell density was greatly decreased as compared to the control (*p* < 0.001; Figure 3D, left panel). Despite the CA1 degeneration, the CA3 cell body layer appeared to be largely preserved in all Voa1 cKO mice examined. The CA3 cell density measures of the Voa1 cKO mice were not significantly different from the controls (*p* = 0.373; Figure 3D, right).

In coronal sections encompassing ventral hippocampal areas (Bregma −3.16 to −3.4 mm), brain hemispheric areas of the Voa1 cKO mice were moderately reduced relative to the controls (*p* = 0.046) but hippocampal areas were not significantly different between the two groups of mice (*p* = 0.284; Figure 3D, right). Ventral hippocampal neurodegeneration was not evident in the Voa1 cKO mice examined (Figures 3A,C). The ventral CA1 and CA3 cell densities of the Voa1 cKO mice were not significantly different from the controls (*p* = 0.784 and *p* = 0.573; Figure 3D, right).

### CA3 Hyperexcitable Activities in Voa1 cKO Mice

**Figure 4:**
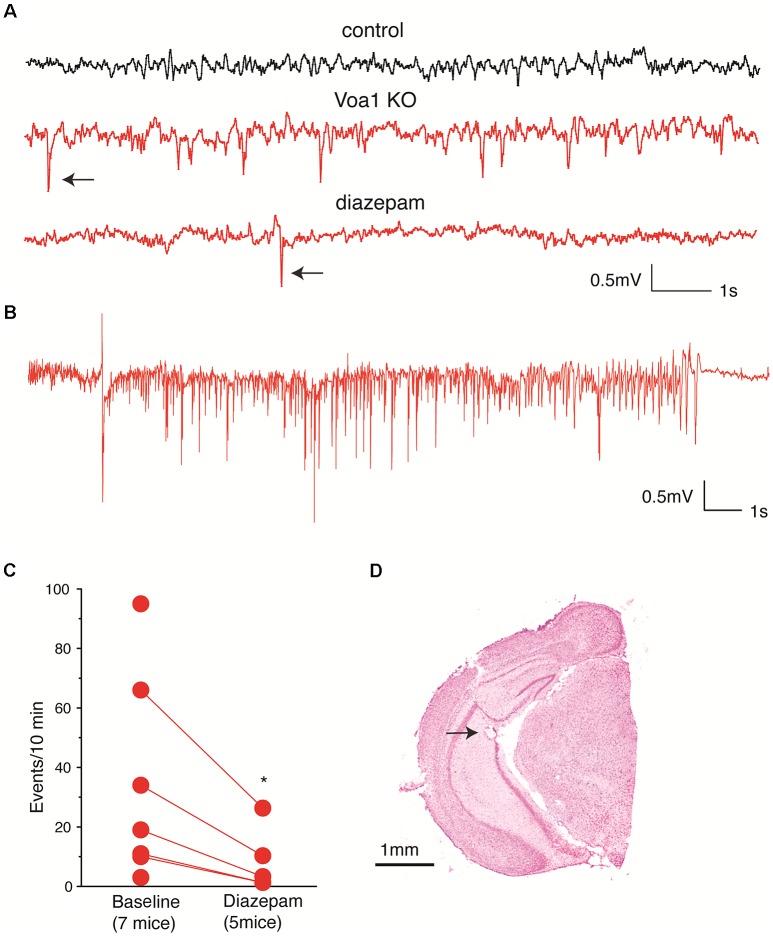
Aberrant CA3 electroencephalographic (EEG) activities observed from the Voa1 cKO mice. **(A)** Representative CA3 EEG traces collected from a control mouse and a Voa1 cKO mouse. Examples of spikes were denoted by filled arrows. Note multiple spikes and a single spike recorded from the Voa1 cKO mouse before and about 2 h following diazepam injection. **(B)** An episode of CA3 discharge collected from another Voa1 cKO mouse. **(C)** Spike rates (events/10 min) measured from seven Voa1 cKO mice. Data collected from five of the seven mice following saline (baseline) and diazepam injections were linked by solid lines. Note a significant decrease in spike rate following the diazepam injections (paired *t*-test). **(D)** A representative cresyl violet-stained section showing the track and putative tip location (filled arrow) of an implanted hippocampal electrode.

The above histological observations motivated us to examine hippocampal CA3 EEG activities from the Voa1 cKO mice. The CA3 recurrent circuitry has intensive projections along the hippocampal longitudinal axis (Witter, 2007) and such CA3 longitudinal network is critical for general “physiological” and “pathophysiological” hippocampal activities (Buzsáki, 2015) but disrupted in brain slices. We anticipated that EEG recordings from the hippocampal CA3 area may have great propensity of revealing aberrant CA3 network activities in the Voa1 cKO mice. Because dorsal hippocampal areas were decreased in the Voa1 cKO mice and it was difficult to target the dorsal hippocampal CA3 in these mice (Figures 3A,D), we implanted electrodes in the middle-ventral hippocampal CA3 area to examine potential aberrant EEG activities. EEG recordings from the hippocampal CA3 area revealed spikes in seven Voa1 cKO mice examined (three female and four male mice; Figures 4A,C,D). These spikes occurred intermittently during behavioral immobility or sleep, and spike rates were variable among individual mice (up to 93 spikes per 10 min; Figure 4C). Diazepam is a benzodiazepine positive allosteric modulator of the GABA_A_ receptors. Previous works from our lab have shown that intraperitoneal injections of diazepam at 1.5 mg/kg were effective in suppressing hippocampal spikes or seizures in other mouse models (El-Hayek et al., 2011, 2013). We thus examined the effects of diazepam in the Voa1 mice. A single intraperitoneal injection of diazepam at 1.5 mg/kg reduced spike rates in five Voa1 cKO mice tested (*p* = 0.026; Figures 4A,C), suggesting that attenuated GABAergic inhibition might partly account for the genesis of aberrant hippocampal spikes in the Voa1 cKO mice.

In addition to the spikes, two discharge episodes were observed from one Voa1 cKO mouse (Figure 4B). These discharges lasted 19–22 s and their occurrence was not associated with evident convulsive behavior. The waveform and behavioral correlate of these discharges were similar to the “non-convulsive electrographic seizure events” previously observed from post-ischemic mice (Song et al., 2018a). In contrast, neither hippocampal spike nor discharge was observed from five control mice examined (two female and three male mice; Figure 4A). Together these EEG observations suggest that the hippocampal CA3 network activity was altered towards hyperexcitability in the Voa1 cKO mice.

## Discussion

We examined the Voa1 cKO mice of ages 5–6 months to determine the persistent effects of Voa1 deletion. Because CaMKα-Cre is expressed in forebrain areas particularly in the hippocampal CA1 (Tsien et al., 1996; Sonner et al., 2005), the focus of our experiments was on structural and functional changes in the hippocampus. The Voa1 knockout mice exhibited evident hippocampal abnormalities. Specifically, the Voa1 cKO mice were impaired in spatial learning and memory as they performed poorly relative to the controls in the visible and hidden platform trials and probe tests of the Morris water maze test. In addition, the Voa1 cKO mice had deficits in hippocampal synaptic functions as the CA1 synaptic potentials recorded in brain slices were greatly diminished. Further, histologically recognized CA1 neurodegeneration was pronounced in the dorsal hippocampus of the Voa1 cKO mice. The CA1 neurodegeneration was associated with general brain atrophy as overall hemispheric areas were reduced in the Voa1 cKO mice relative to the controls. Taking together these observations and the notion that the dorsal hippocampus is crucial for spatial learning and memory (Strange et al., 2014), we postulate that the spatial learning and memory impairment observed from the Voa1 cKO mice may result from the hippocampal CA1 neurodegeneration as well as abnormalities in other brain structures yet to be further characterized. Such CA1 neurodegeneration may largely result from chronic dis-regulations in cellular pH and energy metabolisms due to malfunctions of V-ATPase (Forgac, 2007; Cotter et al., 2015; Hayek et al., 2019). While the exact mechanisms and time course by which CA1 neurons degenerate in the Voa1 cKO mice remain a topic of further investigation, our present observations are in consistent with recent findings that V-ATPase interruption by targeting ATP6AP2 causes neuronal degeneration in other brain regions (Dubos et al., 2015; Fassio et al., 2018; Hirose et al., 2019).

Despite the pronounced CA1 degeneration/dysfunction of the dorsal hippocampus, the hippocampal CA3 neurons appeared to be spared morphologically in the Voa1 cKO mice. In addition, CA3 hyperexcitable activities were observed from the Voa1 cKO mice as CA3 EEG recordings detected aberrant diazepam-sensitive spikes and non-convulsive discharges. These regional differences may mainly result from the discrete Cre expression pattern (Tsien et al., 1996; Sonner et al., 2005) hence the severe Voa1 deletion in the dorsal hippocampal CA1. The vulnerability of the dorsal hippocampal CA1 neurons, which was previously demonstrated in models of brain hypoxia/ischemia (Schmidt-Kastner, 2015), may also be a factor. It is presently unknown as to when and how the CA3 hyperexcitability evolves in the Voa1 cKO mice. One possibility is that the hippocampal CA3 circuitry is altered towards hyperexcitability to compensate the degenerated/dysfunctional CA1 circuitry. Previous studies have demonstrated the hippocampal CA1 degeneration and CA3 hyperexcitability in a rat model of transient global brain ischemia (Wu et al., 2005; Epsztein et al., 2006). When examined a few months after initial ischemic epidotes, rats presented a complete loss of dorsal hippocampal CA1 neurons and robust CA3 EEG spikes or hyperexcitable responses. Together with our present observations and these previous studies raise an intriguing possibility as to whether the CA3 hyperexcitability is a common chronic outcome in rodent models with CA1 neuronal injury/degeneration. Another possibility is that the CA3 hyperexcitability starts prior to or concurs with the CA1 degeneration process hence playing a critical role in the CA1 neurodegeneration through excitotoxicity mechanisms (Lai et al., 2014). As Cre recombinase expression in hippocampus stabilizes after postnatal 19–20 days (Tsien et al., 1996), future experiments that examine the Voa1 cKO mice at different time points during postnatal development may help determine the temporal relation of CA1 degeneration and CA3 hyperexcitability.

Our present experiments have limitations and complications. In particular, the expression of Cre recombinase gene under CaMKIIα promoter caused severe degeneration of dorsal hippocampal CA1 neurons in the Voa1 cKO mice (see above). While the precise timing of CA1 degeneration was unknown, it was difficult for us to investigate whether Voa1 protein is decreased while Voa1 cKO CA1 neurons were still alive using brain sections encompassing dorsal hippocampal areas. Therefore, we decided to use cultured hippocampal neurons as an alternative approach (see “Materials and Methods” section). We infected floxed neurons with lentiviral particles that co-expressed GFP and blasticidin S resistance gene (control) or GFP and Cre recombinase gene (KO) on (DIV 1). As KO neurons started to show signs of deterioration on DIV 13, we harvested control and KO neurons on DIV 13 for Western blot analysis. These experiments demonstrated a clear reduction of Voa1 in KO neurons (Supplementary Figure S1B), suggesting that reduction of Voa1 may take place prior to neurodegeneration. In addition, we used C57BL/6 mice with CaMKIIα-Cre (Tsien et al., 1996) to generate the Voa1 cKO model. Naïve C57 black mice are known to perform well in the Morris water maze test (Wolff et al., 2002; Patil et al., 2009; Kapadia et al., 2016), but experimental factors including the influences by sex and stress responses can substantially affect animal performance in the Morris water maze test (Kapadia et al., 2016). As we used mixed female and male mice in the present experiments, the influences by sex and stress responses on spatial memory outcomes as well as on CA3 EEG signals remain to be evaluated. Moreover, as the Cre recombinase under CaMKIIα promoter is expected to delete Voa1 in forebrain areas in addition to the dorsal hippocampal CA1, structural and/or functional abnormalities in these forebrain areas might also affect the performance of the Voa1 cKO mice in the Morris water maze test and cause other behavioral abnormalities. Future experiments conducting hippocampus-dependent and independent tasks are needed to fully characterize functional deficits in the Voa1 cKO mice. The lack of investigation on molecular and cellular mechanisms of the CA1 degeneration and CA3 hyperexcitability in the Voa1 cKO mice is a major weakness of our present study.

Despite these limitations and weaknesses, the Voa1 knockout caused severe neurodegeneration in the targeted dorsal hippocampal CA1, suggesting that Voa1 related activities are essential for the survival of mammalian central neurons. While severe neurodegeneration makes it impossible for us to examine the precise role of this protein in the process of neurotransmitter release (Saw et al., 2011; Bodzeta et al., 2017), the Voa1 cKO mice may complement other models (Dubos et al., 2015; Fassio et al., 2018; Hirose et al., 2019) for further investigation of V-ATPase dysfunction related neuronal degeneration.

## Data Availability Statement

All data generated or analyzed during this study are included in this published article and are available from the corresponding author on reasonable request.

## Ethics Statement

The animal study was reviewed and approved by the animal care committee of the University Health Network.

## Author Contributions

All authors were involved in experimental design, data discussion and interpretation. Specifically, KM, N-RB, SSh and HH conducted experiments and data analysis. YW and G-H-SW generated and characterized Voa1 antibody. N-RB, SSh, SSu, PM and LZ were responsible for manuscript writing and figure assembling. The submitted manuscript was reviewed and approved by all authors.

## Conflict of Interest

The authors declare that the research was conducted in the absence of any commercial or financial relationships that could be construed as a potential conflict of interest.

## Supplementary Material

FIGURE S1 Generation of Voa1 conditional knockout mice and knockout neurons. **(A)** Top panel, a schematic diagram of the Voa1 floxed allele (labeled as flox) in comparison with wild type (WT) allele; lower panels, genotyping PCR to detect WT vs. floxed allele (left) and CaMKIIα-Cre recombinase gene (right). **(B)** Western blot analysis shows a clear reduction in Voa1 protein in cultured floxed neurons on days *in vitro* 13 (DIV 13) after application of Cre recombinase to the neurons on DIV 1. The level of ubiquitously expressed VCP protein and neuron-specific cytosolic Munc18-1 protein is unchanged.

FIGURE S2 Immunofluorescence images of Voa1 hippocampal expression in wild type mice. Images were obtained from wild type mice of 6 months-old. **Top-left**, a brain section stained with the goat anti-chick antibody alone. Hippocampal subfields and enlarged CA1 and CA3 areas were presented as indicated. **Bottom-left**, another brain section stained with the chicken monoclonal Voa1 antibody IgY and then with the goat anti-chick antibody. Images were similarly presented as in the top panel. **Top-right** and **Bottom-right**, images from same sections showing DAPI staining (blue). Note strong Voa1 signals in hippocampal subfields and enlarged CA1 and CA3 areas in images of the **Bottom-left** panel.
