# Supplementary figures and images for: Observations From a Mouse Model of Forebrain Voa1 Knockout: Focus on Hippocampal Structure and Function
Source: Front Cell Neurosci. 2019 Nov 21;13:484. doi: 10.3389/fncel.2019.00484 (PMC6881385; doi:10.3389/fncel.2019.00484)

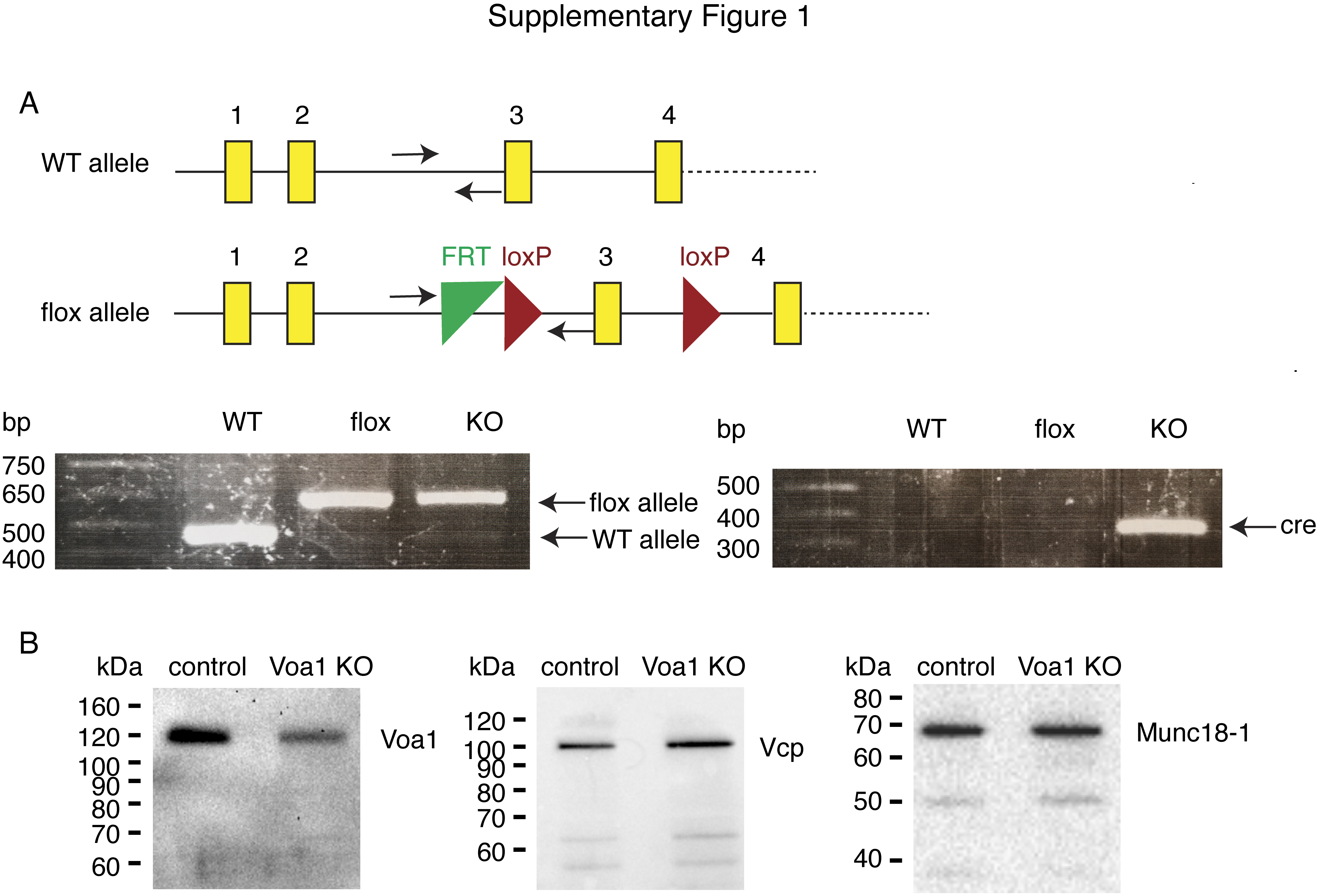

Supplement: FIGURE S1 — Generation of Voa1 conditional knockout mice and knockout neurons. (A) Top panel, a schematic diagram of the Voa1 floxed allele (labeled as flox) in comparison with wild type (WT) allele; lower panels, genotyping PCR to detect WT vs. floxed allele (left) and CaMKIIα-Cre recombinase gene (right). (B) Western blot analysis shows a clear reduction in Voa1 protein in cultured floxed neurons on days in vitro 13 (DIV 13) after application of Cre recombinase to the neurons on DIV 1. The level of ubiquitously expressed VCP protein and neuron-specific cytosolic Munc18-1 protein is unchanged. [file Image_1.TIF]

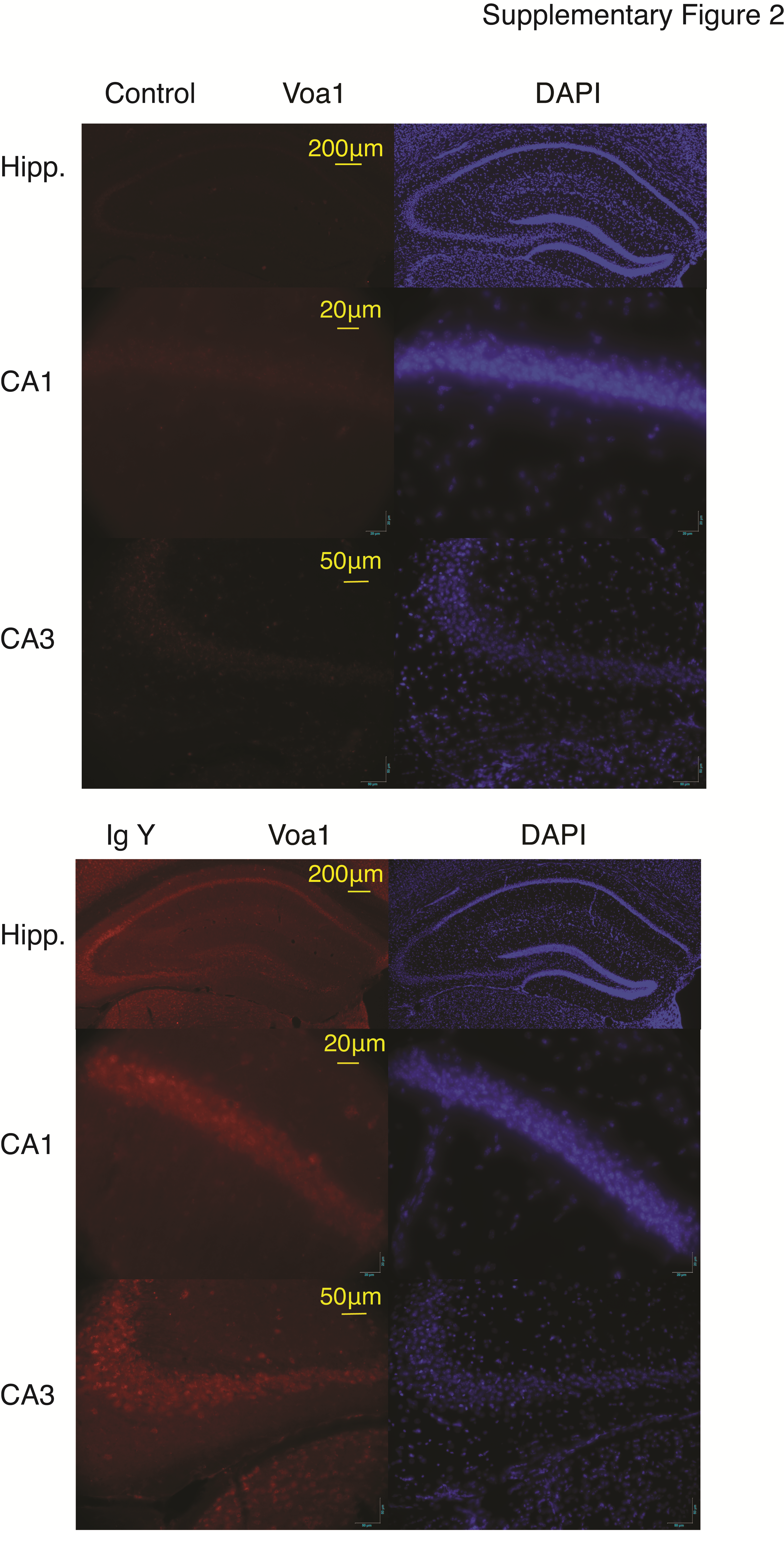

Supplement: FIGURE S2 — Immunofluorescence images of Voa1 hippocampal expression in wild type mice. Images were obtained from wild type mice of 6 months-old. Top-left, a brain section stained with the goat anti-chick antibody alone. Hippocampal subfields and enlarged CA1 and CA3 areas were presented as indicated. Bottom-left, another brain section stained with the chicken monoclonal Voa1 antibody IgY and then with the goat anti-chick antibody. Images were similarly presented as in the top panel. Top-right and Bottom-right, images from same sections showing DAPI staining (blue). Note strong Voa1 signals in hippocampal subfields and enlarged CA1 and CA3 areas in images of the Bottom-left panel. [file Image_2.TIF]
